# Scaffold-Free Engineering of Human Cartilage Implants

**DOI:** 10.1177/19476035211007923

**Published:** 2021-04-15

**Authors:** Nadine Frerker, Tommy A. Karlsen, Magnus Borstad Lilledahl, Sverre-Henning Brorson, John E. Tibballs, Jan E. Brinchmann

**Affiliations:** 1 Department of Immunology, Oslo University Hospital, Oslo, Norway; 2 Department of Physics, Norwegian University of Science and Technology, Trondheim, Norway; 3 Department of Pathology, Oslo University Hospital, Oslo, Norway; 4 Nordic Institute of Dental Materials, Oslo, Norway; 5 Department of Molecular Medicine, University of Oslo, Oslo, Norway

**Keywords:** human articular chondrocytes, chondrogenic redifferentiation, human cartilage implants, cartilage repair, tissue engineering

## Abstract

**Objective:**

Despite new strategies in tissue engineering, cartilage repair remains a major challenge. Our aim is to treat patients with focal lesions of articular cartilage with autologous hyaline cartilage implants using a scaffold-free approach. In this article, we describe experiments to optimize production of scaffold-free cartilage discs.

**Design:**

Articular chondrocytes were expanded *in vitro*, seeded in transwell inserts and redifferentiated using established chondrogenic components. Experimental variables included testing 2 different expansion media, adding bone morphogenetic protein 2 (BMP2), insulin-like growth factor 1 (IGF1), growth/differentiation factor 5 (GDF5), or fibroblast growth factor 18 (FGF18) to the differentiation medium and allowing the disc to float freely in large wells. Cartilage discs were analyzed by weight and thickness, real-time RT-qPCR (reverse transcriptase qualitative polymerase chain reaction), fluorescence immunostaining, transmission electron microscopy, second harmonic generation imaging, and measurement of Young’s modulus.

**Results:**

Addition of BMP2 to the chondrogenic differentiation medium (CDM) was essential for stable disc formation, while IGF1, GDF5, and FGF18 were redundant. Allowing discs to float freely in CDM on a moving platform increased disc thickness compared with discs kept continuously in transwell inserts. Discs cultured for 6 weeks reached a thickness of almost 2 mm and Young’s modulus of >200 kPa. There was abundant type II collagen. Collagen fibrils were 25 nm thick, with a tendency to be organized perpendicular to the disc surface.

**Conclusion:**

Scaffold-free engineering using BMP2 and providing free movement in CDM produced firm, elastic cartilage discs with abundant type II collagen. This approach may potentially be used in clinical trials.

## Introduction

Patients with focal chondral defects can be treated with surgical techniques such as autologous chondrocyte implantation or microfracture. These methods improve functionality and provide pain relief, but result in imperfect tissue repair.^1,2^ Several strategies to redifferentiate chondrocytes in 3-dimensional structures such as pellet cultures or biomaterial matrices have been studied. Pellet cultures serve as a study model but require a large number of cells to generate cartilage constructs of sufficient size.^3^ Biomaterials, on the other hand, are used for the fabrication of scaffolds to be applied in next-generation autologous chondrocyte implantation and tissue engineering. The matrix material is either of biological or synthetic polymeric origin and has to fulfill certain criteria such as being biocompatible and biodegradable while having adequate mechanical properties and scaffold architecture. Drawbacks can be lack of attachment sites for cells, unwanted mechanical properties, insufficient biocompatibility and integrity, or unpredictable biodegradation of the material.^4-6^

An alternative to the implantation of cells within a biomaterial scaffold is the *in vitro* culture of cells spun onto a transwell insert. Here the cells produce their own extracellular matrix (ECM) in a scaffold-free environment. The end product is a disc-shaped tissue containing chondrocytes and their ECM.^7,8^ However, in order to produce ECM molecules that are as similar as possible in type and amount to those found in native articular cartilage, the cells need to be stimulated by the right composition of growth and differentiation factors. Here, we present an approach to optimize the formation of cartilage discs that could potentially be transplanted into cartilage lesions. We have investigated a number of factors that might improve both the process by which the chondrocytes are expanded in cell culture and also the phase during which the chondrocytes are redifferentiated in transwell inserts to produce discs of cartilaginous tissue. Using molecular assays, established and novel imaging techniques, and a testing scheme for biophysical properties we have improved the production of transplantable cartilage discs.

## Methods

### Isolation and Culture of Chondrocytes

All donors provided written, informed consent, and the study was approved by the Regional Committee for Ethics in Medical Research. Osteoarthritis (OA) articular cartilage was obtained from discarded tissue from 6 patients (donors 1-6) with primary OA undergoing knee replacement surgery. As part of the ethical approval of this study, donor demographics were not made available to the scientific team. Cartilage pieces were taken from a part of the surface of the femoral condyle that, by inspection, seemed not to be diseased. Further biopsies were provided from 1 patient undergoing cruciate ligament surgery (donor 7) and 1 patient undergoing meniscus surgery (donor 8). The cartilage tissue was cut and digested as described previously,^9^ then resuspended in DMEM/F12 GlutaMAX medium (Gibco) supplemented with 10% human platelet lysate plasma (hPLP), modified after Schallmoser and Strunk,^10^ with 2 IU/mL heparin (Wockhardt), 10 ng/mL fibroblast growth factor-basic (bFGF; Gibco), 100 units/mL penicillin, and 100 µg/mL streptomycin (P/S; Sigma-Aldrich), and 2.5 µg/mL amphotericin B (Sigma-Aldrich). In some experiments the use of 10% autologous serum^11^ instead of hPLP was tested. The hPLP and autologous serum were sterile filtered prior to use. The cells were expanded in tissue culture plastic flasks, and the culture medium was changed every 3 to 4 days. For passaging, cells at 70% to 90% confluency were trypsinized and passaged at a ratio of 1:3 for further cultivation. Amphotericin B was discontinued after 1 week. Chondrocytes were expanded for 14 days prior submission to chondrogenic redifferentiation. Although some differences were observed between the donors for the chondrocytes’ ability to proliferate *in vitro*, we never failed to have sufficient number of cells for experiments at this time point. Consistent with observations made in our GMP regulated cell production facility (data not shown), addition of bFGF to the cell expansion medium seemed to reduce differences in proliferation capability between donors.

### Chondrogenic Differentiation Medium

The basic chondrogenic differentiation medium (CDM) was DMEM/F12 GlutaMAX supplemented with 10 ng/mL transforming growth factor β1 (TGFβ1; R&D systems), 1% insulin-transferrin-sodium selenite media supplement (Sigma-Aldrich), 0.1 µM dexamethasone (DexaGalen, GALENpharma), 0.1 mM ascorbic acid 2-phosphate (Sigma-Aldrich), 1.25 mg/mL human serum albumin (Octapharma), 4.5 g/L glucose (B. Braun), 40 µg/mL proline (Sigma-Aldrich), 1 mM sodium pyruvate (Gibco), and P/S. Growth factors tested in CDM were 500 ng/mL bone morphogenetic protein 2 (BMP2; InductOs), 100 ng/mL insulin-like growth factor 1 (IGF1; Sigma-Aldrich), 100 ng/mL growth/differentiation factor 5 (GDF5; PeproTech), and 100 ng/mL fibroblast growth factor 18 (FGF18; PeproTech).

### Cartilage Disc Preparation

A total of 0.5 × 10^6^ cells were resuspended in 150 µL of CDM, with or without additional growth factors, and seeded in 6.5-mm polycarbonate transwell inserts in 24-well plates (Corning). The plates were centrifuged for 5 minutes at 200*g* and 700 µL of the CDM to be tested was carefully added to the bottom wells. Medium was changed every second day, including the insert top. Cartilage discs were either grown in transwell inserts (confined cultures), or discs were carefully stripped off the transwell membrane after 10 days and allowed to float freely in medium in 6-well plates, which were placed on a shaker that rotated at 50 to 65 rpm (unconfined cultures). Cartilage discs were harvested after 3 or 6 weeks of chondrogenic redifferentiation. The discs were washed with PBS and further treated as described below.

### Isolation of Total RNA, cDNA Synthesis, and Real-Time RT-qPCR

Cartilage discs were snap-frozen in liquid nitrogen and stored at −80 °C until processing. Frozen discs were crushed in liquid nitrogen with a pestle and total RNA was isolated following the protocol in the miRNeasy mini kit (Qiagen). cDNA synthesis and real-time RT-qPCR (reverse transcriptase qualitative polymerase chain reaction) were performed according to the manufacturer’s instructions of the High Capacity cDNA Reverse Transcription Kit and TaqMan 2x Universal PCR Master Mix (both Applied Biosystems). cDNA samples were probed for cartilage relevant genes using primers from Applied Biosystems. All samples were run in technical triplicates. Glyceraldehyde 3-phosphate dehydrogenase (*GAPDH*) served as endogenous control. Results are shown as expression relative to *GAPDH* using mean values from technical triplicates with a 95% confidence interval.

### Immunofluorescence Analysis

Cartilage samples were embedded in Frozen Section Medium (Richard-Allan Scientific Neg50, Thermo Scientific) and frozen in dry ice-cooled isopentane. Frozen tissue blocks were stored at −80 °C. The samples were cut in 9- to 10-µm thick sections on a CryoStar NX70 Cryostat (Thermo Scientific), mounted on SuperFrost Plus Adhesion slides, stored at −80 °C and fixed for 60 seconds in cold 95% ethanol directly before starting immunostaining. Sections were immunostained for the presence of type II collagen (COL2; clone II-4C11; MP Biomedicals; at 0.833 µg/mL), aggrecan (ACAN; clone 4F4; Santa Cruz; at 0.1 µg/mL), type I collagen (COL1; clone EPR7785; Abcam; at 0.8 µg/mL), type X collagen (COL10; clone X53 diluted 1:200; generous gift from Prof. Klaus von der Mark). Antibodies were diluted in 1.25% BSA in PBS, and slides were incubated at 4 °C overnight. Negative controls were made by omitting the primary antibody. The secondary antibodies, goat anti-mouse IgG conjugated to Alexa 594 and goat anti-rabbit IgG conjugated to Alexa 488 (both Life Technologies), were diluted 1:400. The stained sections were mounted with ProLong Gold antifade reagent (Invitrogen), containing DAPI for nuclear staining. Imaging was done using an upright Nikon Eclipse E600 microscope equipped with an Olympus ColorView III camera. Thickness of cartilage discs was measured using histological sections.

### Phalloidin Staining

Chondrocytes at day 17 of culture were seeded on coverslips in hPLP/bFGF-supplemented culture medium. After 24 hours the cells were carefully washed and medium was exchanged with CDM with or without BMP2. After further 24 hours of differentiation cells were fixed with 4% paraformaldehyde for 15 minutes at room temperature, permeabilized in 0.1% Triton X-100 for 3 to 5 minutes and labeled with phalloidin (Phalloidin-iFluor 488 Reagent, ab176753, Abcam) according to the manufacturer’s protocol. Coverslips were mounted on microscope slides using Fluoroshield with DAPI (Sigma). Fluorescent images were taken using an upright Nikon Eclipse E600 microscope equipped with an Olympus ColorView III camera. Light microscopy images were taken before staining of samples using a Zeiss Axio Vert.A1 inverted microscope equipped with a Zeiss AxioCam MRm camera.

### Mechanical Testing

The cartilage discs were subjected to displacement-controlled mechanical testing in an electrodynamic instrument (Bose Electroforce 3300, TA Electroforce) equipped with a temperature controlled water chamber set to 35 ± 0.1 °C. An inner reactor attached to the lower platen of diameter 25 mm was filled with PBS. The upper, 23 mm diameter platen was controlled first manually to detect contact with the specimen and then by program with the test sequence. During each test, displacement and force were sampled at 100 Hz, to precisions of 1 µm and 0.3 N, respectively.

From detected contact, the test sequence compressed the specimen by a nominal 15% in 0.5 seconds and held 30 seconds, before increasing compression to 30% over 30 seconds. The latter compression was repeated after rapid decompression to 15% followed by a 30-second hold. The elastic modulus was calculated for compression between 15% and 30%.

### Transmission Electron Microscopy

The tissue was fixed in a mixture of 2% glutaraldehyde and 0.5% paraformaldehyde in cacodylate buffer for 24 hours at 4 °C. Afterwards the tissue was post-fixed in 2% osmium tetroxide for 2 hours at 4 °C and further dehydrated, infiltrated, and embedded in epoxy resin (Epon). After polymerization, semi-thin sections were cut and stained with toluidine blue in order to localize the region of interest for making ultrathin sections. The epoxy blocks were then trimmed with respect to the structure of interest, and 70-nm ultrathin sections were cut on an ultramicrotome (Leica, UPC6) followed by staining with 4% uranyl acetate in 40% ethanol and Reynolds’ lead citrate. Finally, the ultrathin sections were examined in a transmission electron microscope (Tecnai12, FEI).^12^

### Second Harmonic Generation Microscopy

Samples were fixed in 4% formalin and embedded in paraffin before being cut (4 µm) and mounted on glass slides. The sections were deparaffinized and rehydrated using standard laboratory procedures and finally mounted with ProLong Gold antifade, containing DAPI. Second harmonic generation (SHG) microscopy was performed on a Leica SP8 confocal imaging system equipped with a Coherent Vision S femtosecond laser tuned to 890 nm for SHG from collagen and for 2-photon excitation of DAPI. SHG was detected using a non-descanned transmitted light detector (conventional PMT) with a 435- to 455-nm bandpass filter. DAPI was collected with a non-descanned reflected light detector (Hybrid PMT) with a 500- to 550-nm bandpass filter. Images were collected with a 25× objective with numerical aperture of 0.9. A 2D Gaussian filter with a 0.5 standard deviation of 0.5 and a 2D median filter with a 3 × 3 kernel was used to reduce noise in both channels. A gamma of 0.5 was applied to the SHG channel to enhance the visibility of the lower intensities.

## Results

### Addition of BMP2 but Not IGF1 Improved Cartilage Disc Formation in 3-Week Confined 3D Cultures

**Figure 1.**
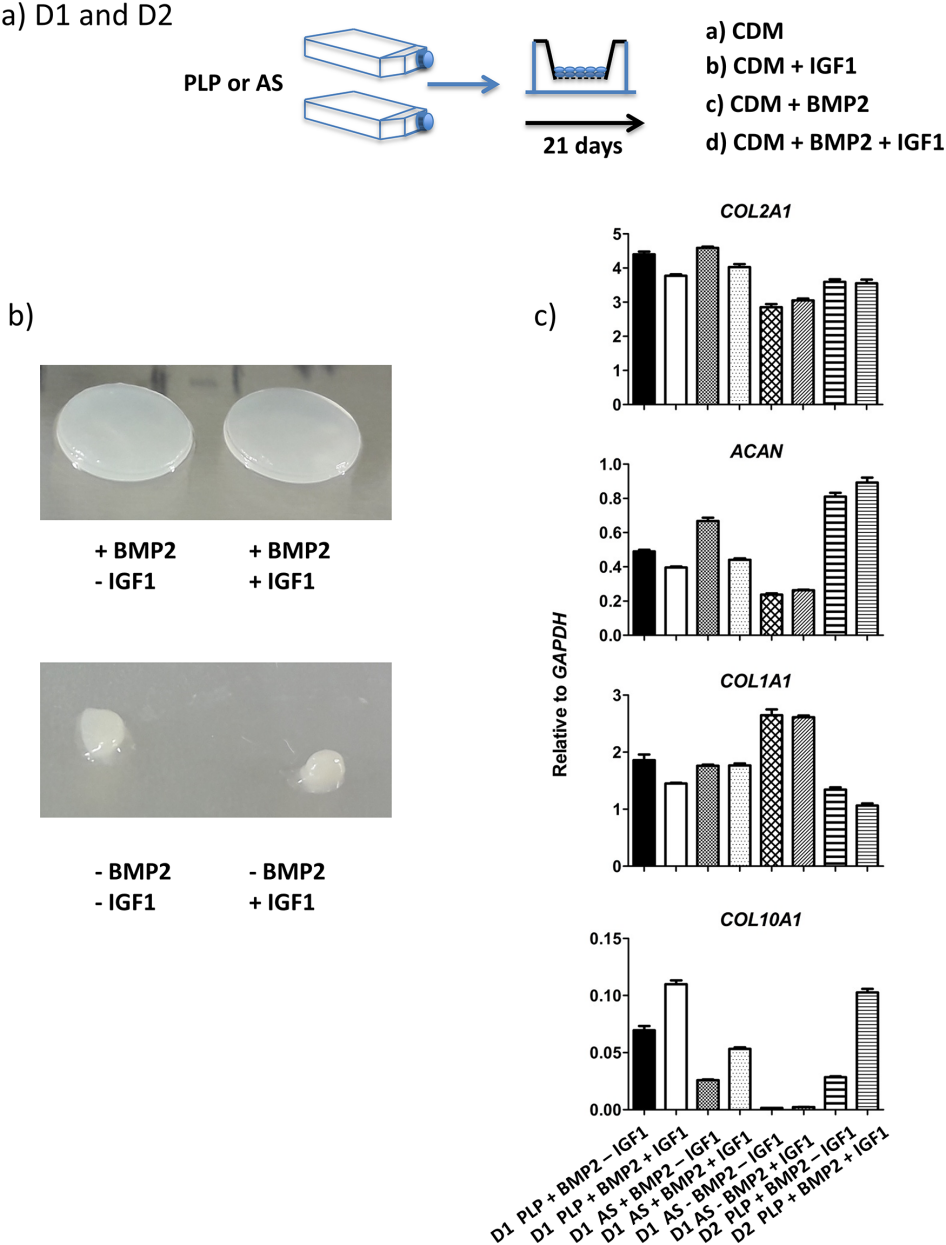
(**a**) OA chondrocytes from donor 1 (D1) and donor 2 (D2) were expanded in bFGF-supplemented medium containing either hPLP or autologous serum (AS) and redifferentiated for 3 weeks in transwell inserts (confined) in the presence or absence of BMP2 and/or IGF1. (**b**) Examples showing cartilage constructs from cells expanded in hPLP (images with same scale). Chondrocytes formed solid discs in the presence but not in the absence of BMP2, independent of supplementation with IGF1. (**c**) Real-time RT-qPCR. Expression of *COL2A1*, *ACAN*, *COL1A1*, and *COL10A1* mRNA. No data for D2 expanded in serum are available.

In our first series of experiments we wanted to test the impact of the known anabolic factors BMP2 and IGF1 on cartilage disc formation in 3-week transwell cultures. Chondrocytes were cultured in the presence of bFGF in medium containing either hPLP or autologous serum. Cells were then redifferentiated in CDM alone, CDM supplemented with either BMP2 or IGF1 or in CDM with both factors (**Fig. 1a**). Cartilage samples were analyzed by visual inspection and real-time RT-qPCR on day 21. Cells expanded in hPLP and then redifferentiated on transwell membranes in the absence of BMP2 aggregated to form pellets, regardless of the absence or presence of IGF1 in the differentiation medium (**Fig. 1b**). An additional experiment exposing chondrocytes to either CDM alone or CDM supplemented with BMP2 was performed. BMP2-induced changes in cell morphology or apparent differences in the actin cytoskeleton^13^ could not be revealed by phalloidin staining (Suppl. Fig. S1). Cells expanded in medium supplemented with autologous serum and then redifferentiated in the absence of BMP2 usually attached to the membrane, but the discs were thin and frail. Cells redifferentiated in transwell inserts in the presence of BMP2 always made robust cartilage discs.

Failure to form discs is the reason why real-time RT-qPCR data from hPLP-expanded cells redifferentiated without BMP2 are absent from **Fig. 1c**. There were minor differences in the levels of mRNA expression of *COL1A1*, *COL2A1*, *ACAN*, and *COL10A1* between the different conditions. However, absence of BMP2 reduced the expression of *COL10A1* mRNA in discs made by cells expanded in medium supplemented with autologous serum, and IGF1 tended to increase the expression of *COL10A1* (**Fig. 1c**).

Based on these observations, CDM supplemented with BMP2 was used in all future experiments and called CDMB.

### Improved Disc Thickness under Unconfined Differentiation Conditions

**Figure 2.**
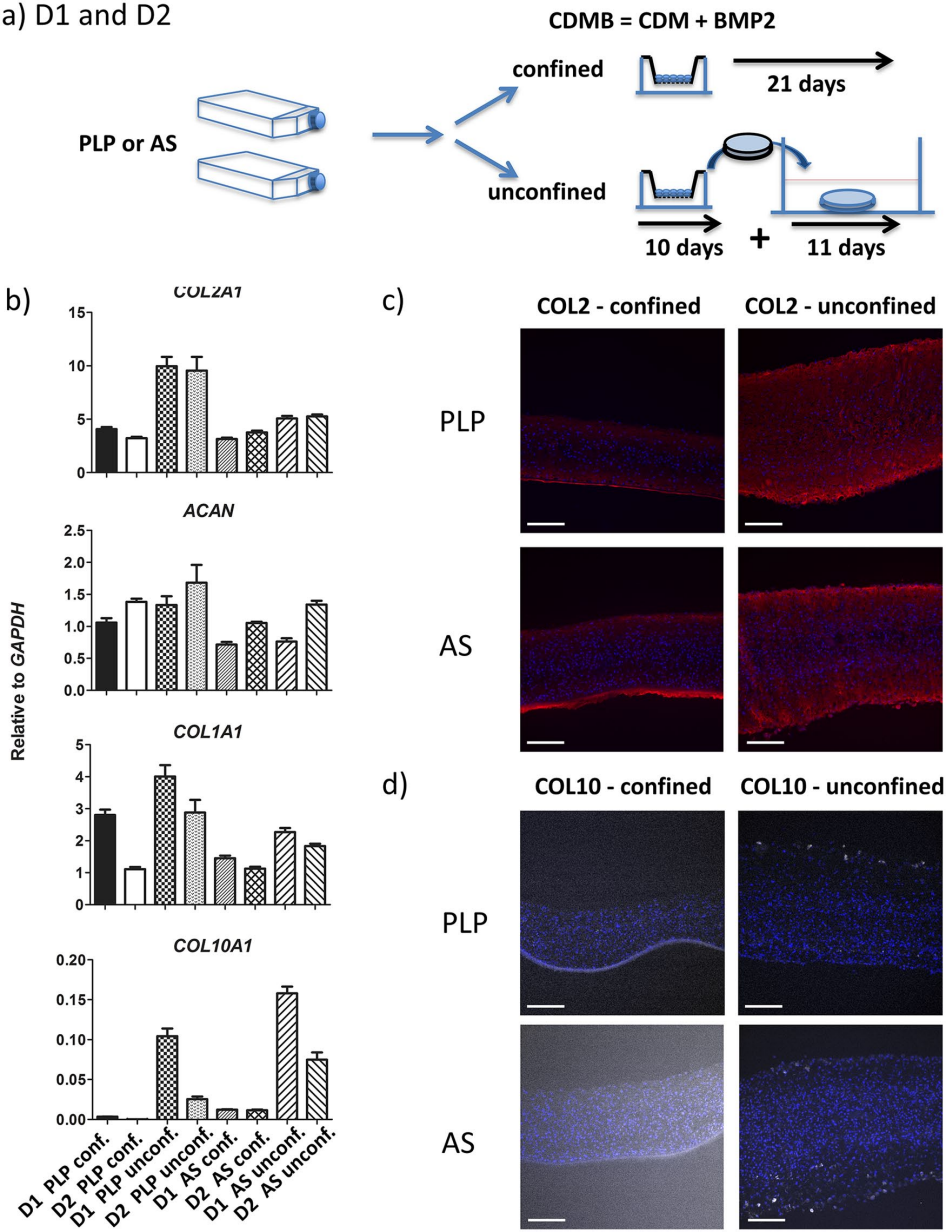
(**a**) OA chondrocytes from D1 and D2 were expanded in bFGF-supplemented medium containing either hPLP or AS and redifferentiated for 3 weeks under confined or unconfined conditions. (**b**) Real-time RT-qPCR. Expression of *COL2A1*, *ACAN*, *COL1A1*, and *COL10A1* mRNA. Immunofluorescence of (**c**) COL2 (red) and (**d**) COL10 (white) expression in confined and unconfined cartilage discs after 3 weeks of redifferentiation. Examples from chondrocytes expanded in either hPLP- or AS-supplemented medium. Nuclear staining with DAPI is presented in blue color. Sections of confined cartilage discs include the membrane. Scale bar = 200 µm.

Seeding 0.5 × 10^6^ actively proliferating cells in an insert placed within a small well limits the volume of medium and thus of nutrients available for the chondrocytes. To possibly improve on this, we established cultures where the discs were stripped off the membranes on day 10 of chondrogenic redifferentiation and allowed to float freely in slowly shaken 6-well plates — unconfined condition (**Fig. 2a**). This approach was adapted and modified from Anderson *et al*.^7^ Unconfined conditions produced discs with approximately double thickness compared with confined conditions on day 21 (**Table 1**). The expression of cartilage relevant genes did not consistently parallel the difference in disc thickness (**Fig. 2b**).

**Table 1.** Thickness of Cartilage Discs.

Expansion Condition	Mode of Chondrogenic Redifferentiation	Thickness (nm)
Donor 1		
hPLP	Confined	306
hPLP	Unconfined	640
Serum	Confined	381
Serum	Unconfined	720
Donor 2		
hPLP	Confined	340
hPLP	Unconfined	760
Serum	Confined	412
Serum	Unconfined	760

hPLP = human platelet lysate plasma.

Staining of frozen sections for COL1, COL2, ACAN, and COL10 gave semiquantitative results for the presence of ECM molecules. As shown in **Fig. 2c**, unconfined cartilage discs tended to give stronger COL2 staining compared with confined discs. In contrast to confined discs, where COL10 positive cells could not be seen, unconfined discs revealed intracellular COL10 expression in a few cells (**Fig. 2d**).

At the end of these experiments the expansion medium was chosen to be hPLP supplemented with bFGF. Autologous serum with bFGF gave similarly good results, but required inconveniently large amounts of blood from the donor in order to obtain large numbers of chondrocytes. Based on disc thickness in combination with trends in real-time RT-qPCR and staining analyses, unconfined differentiation culture conditions were chosen over confined conditions. For the following experiments inserts were transferred from 24-well plates to 6-well plates on day one of differentiation culture using custom-made adaptors.

### Differentiation of Chondrocytes in the Presence of GDF5, FGF18, or Short-Term Exposure to TGFβ1 Did Not Increase Cartilage Disc Quality in 6-Week Unconfined Cultures

**Figure 3.**
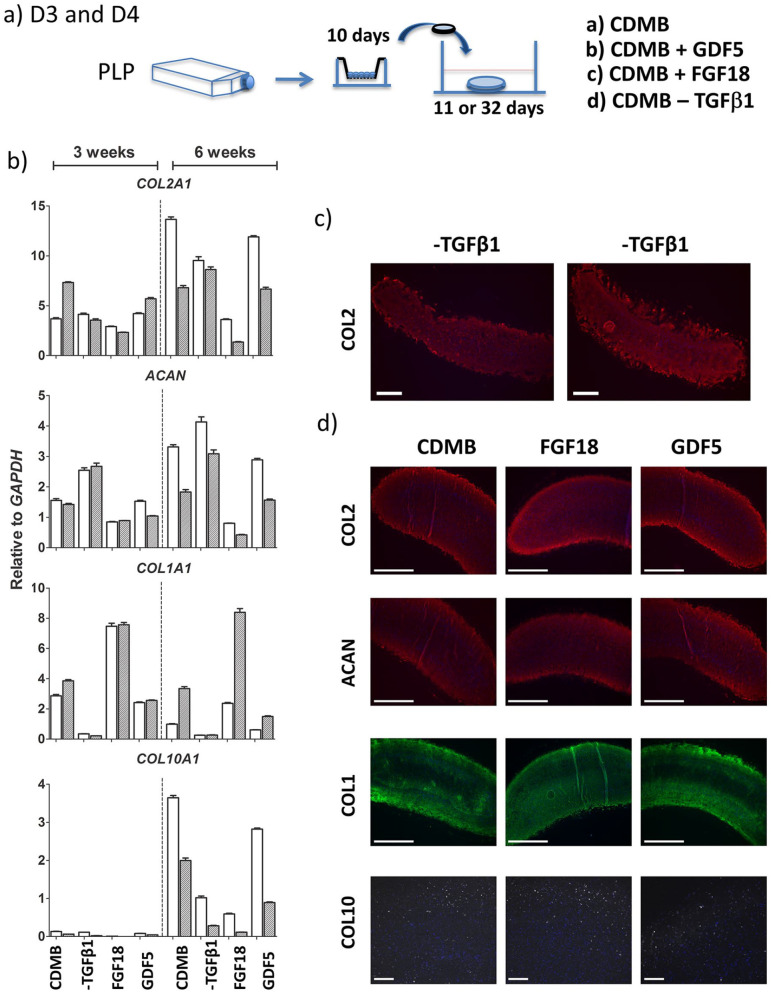
(**a**) OA chondrocytes from D3 and D4 were expanded in bFGF-supplemented medium containing hPLP and redifferentiated for 3 and 6 weeks under unconfined conditions in differentiation medium CDMB (control) or CDMB supplemented with FGF18 or GDF5, or with TGFβ1-withdrawal after 3 days of exposure. (**b**) Real-time RT-qPCR. Expression of *COL2A1, ACAN, COL1A1*, and *COL10A1* mRNA shown for 2 donors (white and grey bars). (**c**) Immunofluorescence staining of cartilage discs after 6 weeks of redifferentiation. COL2 expression in samples exposed to TGFβ1 for only 3 days (−TGFβ1). Example from 2 different donors. Scale bar = 500 µm. (**c**) Discs redifferentiated in CDMB, CDMB + FGF18, and CDMB + GDF5 stained for COL2 (red), ACAN (red), COL1 (green; scale bar = 1000 µm), and COL10 (white; scale bar = 200 µm) expression. Nuclear staining with DAPI is presented in blue color.

To further enhance the CDMB cocktail, we next tested the influence of other growth factors in the disc differentiation model in a new set of donor cells in the following parallel experiments: (1) CDMB (control), (2) CDMB supplemented with 100 ng/mL GDF5,^14^ (3) CDMB where TGFβ1 was withdrawn after 72 hours, and (4) CDMB supplemented with 100 ng/mL FGF18^15^ between days 7 and 14 (**Fig. 3a**). The results of real-time RT-qPCR analyses are shown in **Fig. 3b**. Compared with CDMB alone, addition of GDF5 or FGF18 gave similar or lower levels of *COL2A1* and *ACAN* both at 3 and 6 weeks. The levels of *COL1A1* were consistently higher when FGF18 was added to the differentiation mixture, while the results when GDF5 was added were similar or slightly lower compared with CDMB alone. *COL10A1* mRNA was expressed at very low levels at 3 weeks in all conditions of differentiation. However, at 6 weeks CDMB gave the highest *COL10A1* levels. Transient supplementation with TGFβ1 for only 3 days gave similar or slightly higher levels of *COL2A1* and *ACAN* compared with supplementation throughout the differentiation culture in CDMB, and considerably lower levels of *COL1A1* and *COL10A1* mRNA.

Measurement of disc thickness showed that CDMB with or without FGF18 supplementation yielded similar results, CDMB with GDF5 supplementation gave thinner discs, and CDMB with only transient TGFβ1 supplementation even thinner discs (**Table 2**). Measurement of disc wet weight gave similar results, with transient TGFβ1 supplementation giving the lowest weights (**Table 2**).

**Table 2.** Wet Weight and Thickness of Cartilage Discs in Unconfined Culture.

Medium Condition	Donor 3			Donor 4		
	Weight (mg)		Thickness (mm)	Weight (mg)		Thickness (mm)
	3 weeks	6 weeks	6 weeks	3 weeks	6 weeks	6 weeks
CDMB	42.7	95.1	1.79	50.7	103.1	1.82
−TGFβ1	38.8	67.7	1.07	30.5	60.3	1.32
FGF18	50.4	114.3	1.93	46.6	90.3	1.79
GDF5	41.3	92.9	1.46	45.5	102.8	1.67

Immunofluorescence analysis further confirmed that transient TGFβ1 supplementation was inferior to persistent TGFβ1 in the CDMB: the discs were thin with fragile tissue at the edges for both donors (**Fig. 3c**). Immunostaining of discs made under the other 3 conditions gave similar staining for COL2, COL1, and ACAN (**Fig. 3d**). Again, COL10 was present only as intracellular protein, expressed in some cells in the periphery of the disc (**Fig. 3d**). As supplementation of CDMB with additional growth factors did not result in significant improvements, future experiments were carried out with CDMB only.

### Imaging Analyses and Mechanical Testing

**Figure 4.**
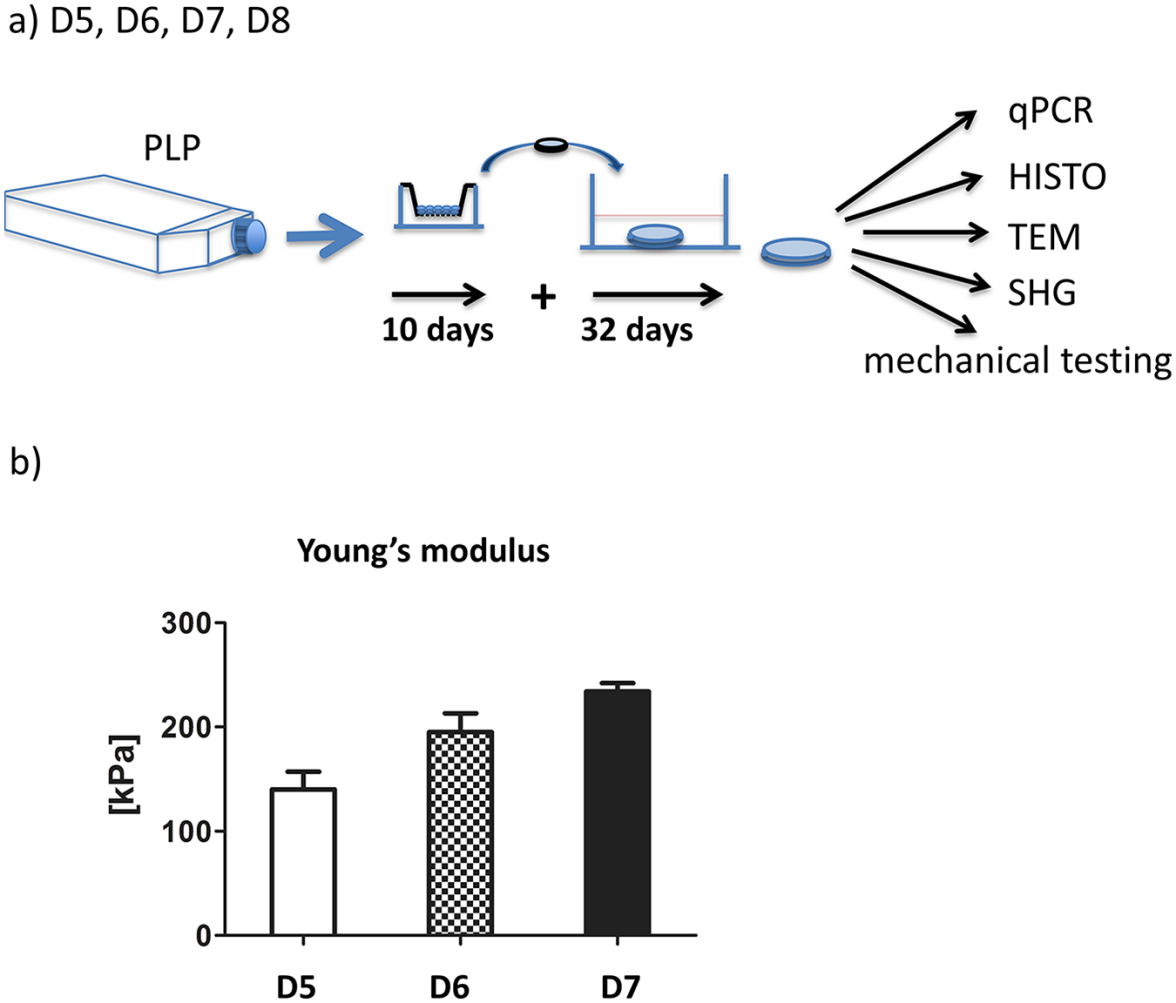
(**a**) Chondrocytes from D5, D6, D7, and D8 were expanded in bFGF-supplemented medium containing hPLP and redifferentiated for 6 weeks under unconfined conditions in differentiation medium CDMB. Samples were analyzed by real-time RT-qPCR, immunofluorescence, TEM, SHG microscopy, and mechanical testing. (**b**) Young’s modulus of cartilage discs from 3 donors. Measurements were performed unconfined at 35 °C in PBS.

Using CDMB and redifferentiated chondrocytes from another 4 donors we went on to examine the cartilage discs for mechanical properties and appearance using other imaging modalities (**Fig. 4a**). Histology, disc weight, and gene expression did not show significant differences between samples and was similar to the 6-week cartilage discs grown in CDMB of the previous experiment (donors 3 and 4). At 6 weeks the ratio of *COL2A1*/*COL1A1* mRNA was median 26.7 (range 17.0-55.1) for donors 5 to 8 while the ratio for *COL2A1*/*COL10A1* was median of 20.0 (range 6.5-24.0).

Young’s modulus was similar for the 3 tested samples with values between 140 kPa and 234 kPa (**Fig. 4b**). The concavity of the disc surface from donor 8 prevented the calculation of Young’s modulus of this disc.

**Figure 5.**
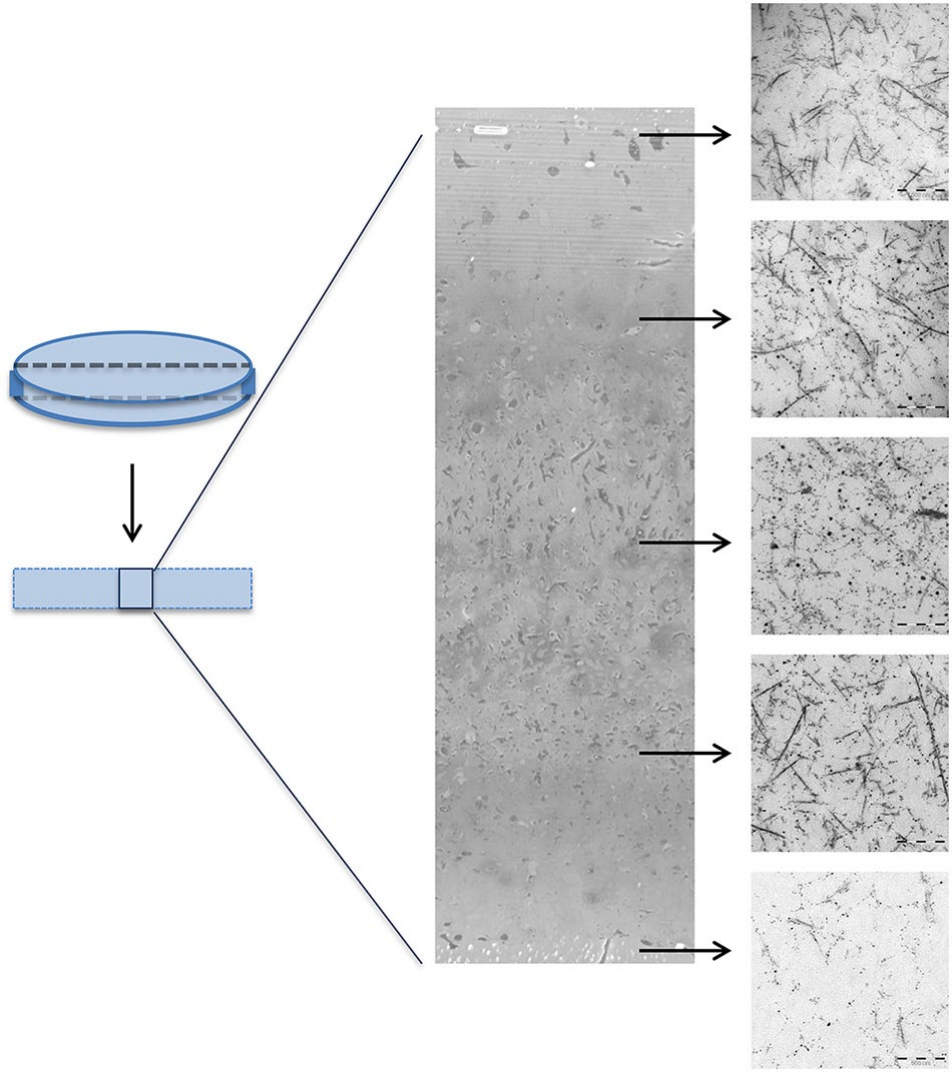
Transmission electron microscopy. Example shown from one donor (donor 5). The presented selection is a frontal sample from the middle of the cartilage disc (left). Cross section of the cartilage disc (center) showing sites of TEM images of the matrix (right, scale bar = 500 nm).

A comparison of cartilage discs using transmission electron microscopy (TEM) showed the formation of collagen fibrils throughout the sections of cartilage samples and supported the similarity between discs (**Fig. 5**, Suppl. Fig. S2). In general, more dead cells were observed in the central region. The fibrils showed a thickness of approximately 25 nm.

**Figure 6.**
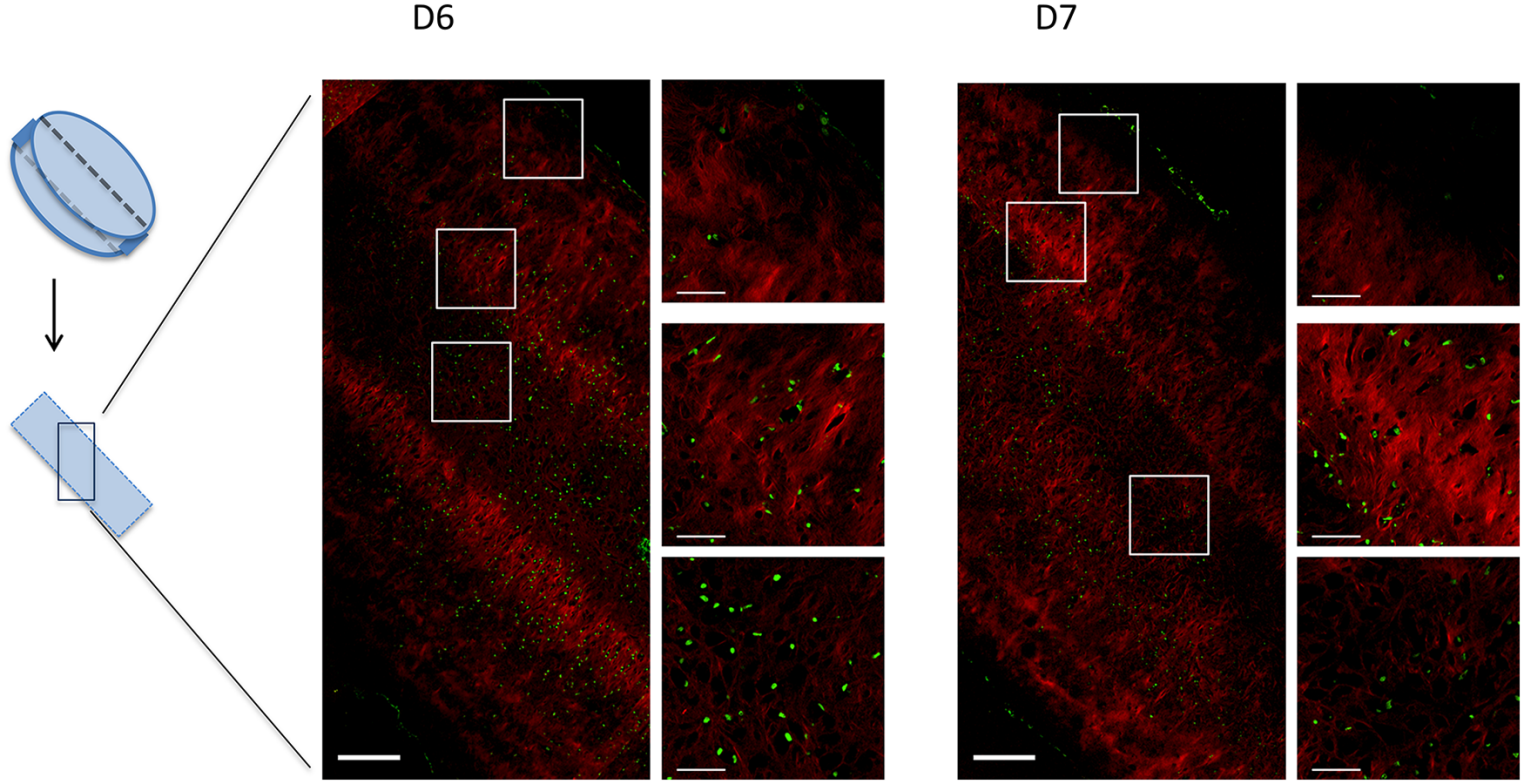
Second harmonic generation microscopy. Examples from cartilage discs based on chondrocytes from 2 donors (donors 6 and 7). The section (left) is a frontal sample from the middle of the cartilage disc at low magnification (scale bar = 200 µm) showing sites of selected high-resolution images (right, scale bar = 50 µm). Fibrillar collagen presents in red, nuclear staining with DAPI presents in green.

SHG microscopy was also used for detection of fibrillar collagen. Representative pictures of the SHG signal from cartilage disc sections showed some variation in structure and density across thickness (**Fig. 6**, Suppl. Fig. S3). In general, the pattern revealed a layer with less dense collagen in the center of the disc accompanied by layers with denser collagen on either side. SHG suggested that many fibrils were oriented perpendicular to the disc surface.

Taken together, our results show cartilage discs with a strong presence of COL2, with a disc height approximately that of articular cartilage in adult ankles and with mechanical properties approaching but not similar to human articular cartilage.

## Discussion

Despite new materials and approaches in tissue engineering, replacement of native articular cartilage with its unique functional properties remains a major challenge in cartilage repair. Using autologous chondrocytes and a scaffold-free approach, biocompatibility problems and potential limitations introduced by a synthetic scaffold are avoided. The aim of this study was to establish and to optimize a reliable model of scaffold-free cartilage disc production for potential application in the clinic using human articular chondrocytes. Simple modifications to the basic model, such as using unconfined culture conditions and adding BMP2 to the differentiation medium, allowed for the engineering of almost 2-mm-thick cartilage discs in 6 weeks of 3-dimensional culture. The discs contained abundant COL2, collagen fibrils 25 nm in width with a gross orientation perpendicular to the disc surface, and were shown to have a Young’s modulus of approximately 200 kPa. Although more research is required to make perfect hyaline cartilage in the laboratory, we believe these improvements bring engineered cartilage discs closer to the quality required for therapeutic transplantations.

In our first set of experiments we expanded chondrocytes in medium containing either hPLP or autologous serum. According to GMP guidelines, animal products should be replaced by human alternatives for clinical translation.^16^ Both hPLP and autologous serum turned out to be good options for chondrocyte expansion. However, for research-based experiments hPLP is the more convenient alternative as it can be used as an off-the-shelf product. The large number of donors included in the plasma and platelet lysate pools also reduces the impact of patient-to-patient variability.

We tested supplementation of CDM with BMP2 and IGF1. The anabolic growth factor IGF1 is involved in cartilage homeostasis^17^ and BMP2 is relevant for cartilage development and matrix production.^18-21^ IGF1 did not improve outcome in our cartilage disc assay, and tended to increase the levels of cartilage hypertrophy marker *COL10A1* mRNA. A similar increase has been observed in another chondrocyte culture model.^22^ The addition of BMP2 to CDM, referred to as CDMB, turned out to be of major importance for reliable disc formation in our model: while discs cultured without BMP2 either collapsed into small pellets or gave thin and fragile discs, the addition of BMP2 robustly gave discs with a thickness approaching that seen in adult weight-bearing joints. BMP2 is not routinely used in chondrogenic differentiation media, and omission of BMP2 might be the reason for the disc collapse reported by groups using uncoated inserts.^23,24^ Since no discs could be formed in the absence of BMP2, its effect on the actin cytoskeleton was tested in chondrocytes grown in monolayer. Phalloidin staining did not show clear differences. However, an impact of BMP2 might possibly be observed in 3D. Interestingly, adding BMP2 to CDM did not impact importantly on the mRNA expression of chondrogenic genes *COL2A1* and *ACAN*, but greatly increased the production of cartilage ECM. Similar observations have been made using human mesenchymal stromal cells (hMSCs).^20,24^ The mechanism regulating this effect has not been fully investigated, although a study using mouse chondrocytes suggests that BMP2 may impact on the type of procollagen produced.^25^ Although the use of BMP2 has been related to hypertrophy of chondrocytes and calcification of cartilage ECM associated with increased expression of COL10 mRNA and protein,^26-28^ Payr *et al*. have argued that hypertrophy and expression of osteogenic markers after BMP2 stimulation might be more relevant when using MSCs than human articular chondrocytes.^29^ Still, the role played by BMP2 in the CDMB deserves further investigation.

With respect to IGF1, a study on the redifferentiation capacity of human chondrocytes in pellet cultures has shown that TGFβ1 alone had positive effects on expression of ECM components under normoxia, whereas a positive effect of IGF1 in combination with TGFβ1 was only seen under hypoxic conditions.^30^ In this context, beneficial effects of IGF1 in our experiments might not be detectable under normoxic conditions. Another possibility could be impaired responsiveness to IGF1, which has been reported for aged^31^ and for osteoarthritic chondrocytes.^32^

Furthermore, we compared 2 different approaches of cartilage disc formation — confined and unconfined culture conditions. The latter approach resulted in increased thickness of the discs. Liberation of the freshly formed discs from transwell inserts presumably gives a better supply of nutrients from all sides of the disc and a more stable environment due to a greater volume of the culture medium. Additionally, the slight movement of unconfined discs might contribute to matrix production, which is in accordance with the observation that mechanical stimulation is beneficial for cartilage formation and cartilage tissue engineering.^33-35^

In addition to BMP2 and IGF1, we also tested other redifferentiation cocktail ingredients that might improve cartilage disc formation. FGF18^15^ and GDF5^14^ have been reported to contribute to matrix production and chondrogenesis, but did not show beneficial effects in our experiments. Murphy *et al*. suggested that supplementation of their CDM with GDF5 in addition to TGFβ1 and BMP2 reduced COL1 synthesis in articular chondrocytes.^14^ Numerical values for *COL1A1* mRNA levels were lower also in our experiments when GDF5 was added, but we consider the differences to be too small to be relevant. Speaking against addition of GDF5 was also a slightly lower disc thickness. As FGF18 gave higher *COL1A1* mRNA values in the one experiment, we have so far concluded that FGF18 supplementation does not give a clear beneficial effect. However, it is possible that intermittent exposure during redifferentiation might yield more obvious positive effects, perhaps also reducing *COL1A1* levels.^15^ Finally, based on a study which showed that TGFβ1 withdrawal from chondrogenic differentiation cultures is related to greater cell proliferation and/or survival and that sustained exposure was not essential for proteoglycan synthesis,^36^ we also wanted to check whether short-term exposure to TGFβ1 in CDMB might be sufficient to drive chondrogenesis. Interestingly, mRNA levels of *COL2A1* and *ACAN* were as good or better than for sustained exposure to TGFβ1, while mRNA values for unwanted molecules *COL1A1* and *COL10A1* were lower. However, the discs themselves were thin and fragile, making this approach unacceptable for our project. Formation of fragile tissue in the absence of TGFβ has also been observed by others.^37^

Having chosen the expansion medium and differentiation conditions, we finally evaluated the cartilage discussing TEM and SHG imaging and mechanical testing for stiffness. TEM and SHG microscopy both confirmed the formation of collagen fibrils. By TEM, the thickness of the fibrils was measured to be 25 nm. This is similar to the small prototypic collagen fibrils found in human articular cartilage, and measured to be 18 ± 5 nm thick.^38^ By SHG microscopy, the orientation of the fibrils were found to be grossly perpendicular to the disc surface, which is similar to the orientation of collagen fibrils in the deep zone of articular cartilage.^39^ The values obtained for Young’s modulus were lower than those obtained for native articular cartilage,^40-42^ but comparable to those published previously for discs made from hMSC.^43^ We speculate that it may be difficult to obtain much higher values for tissue stiffness in the absence of periodic loading similar to that exerted on articular cartilage during walking.

Since we wanted to test many parameters (different expansion media, confined vs. unconfined differentiation conditions, several differentiation factors) using different assays, we have tested only 2 donors for the first series of experiments. As this precludes the use of statistics, we only describe very robust observations as important such as the use of unconfined differentiation cultures and the importance of BMP2 for matrix production. Had we wanted to identify less important differences, using biological replicates, we would have had to include a very large number of donors. At this stage we believe such differences would not impact greatly on the end product: transplantable autologous cartilage discs. For the second set of experiments, chondrocytes from 4 donors were used, of which 2 donors had been operated for insertion of prosthesis due to OA, while the other 2 donors were operated for non-OA conditions. Acknowledging that the sample size and number of assays are small, we found that disc thickness was at least as large in discs made from OA chondrocytes, and we saw no discernable differences in any of the other assays presented here between discs made from OA and non-OA chondrocytes.

## Conclusion

Here we present an improved scaffold-free method of reliable cartilage formation using articular chondrocytes that results in discs with a thickness and stiffness approaching those of the articular cartilage of knees and ankles.^44,45^ Our research shows that BMP2 is essential for robust disc formation and that unconfined conditions for the differentiation culture greatly improved matrix production. The discs contained abundant type II collagen and fibrils that tended to be oriented perpendicular to the disc surface. These results suggest that therapeutic trials using scaffold-free cartilage disc implants may be possible in the not too distant future.

## Supplemental Material

sj-pdf-1-car-10.1177_19476035211007923 – Supplemental material for Scaffold-Free Engineering of Human Cartilage Implants Click here for additional data file. Supplemental material, sj-pdf-1-car-10.1177_19476035211007923 for Scaffold-Free Engineering of Human Cartilage Implants by Nadine Frerker, Tommy A. Karlsen, Magnus Borstad Lilledahl, Sverre-Henning Brorson, John E. Tibballs and Jan E. Brinchmann in CARTILAGE
