# Supplementary material for: Scaffold-Free Engineering of Human Cartilage Implants
Source: Cartilage. 2021 Apr 15;13(1 Suppl):1237S–1249S. doi: 10.1177/19476035211007923 (PMC8725371; doi:10.1177/19476035211007923)

Figure legends supplementary data

Figure S1 Phalloidin staining. Cells after 24 h exposure to CDM with BMP2 (left) or without BMP2 (right). Light microscopy images of cells directly before staining (top, scale bar = 200 µm) and fluorescence microscopy of cells after staining with fluorescent phalloidin presented in green color (bottom, scale bar = 100 µm). Nuclear staining with DAPI is presented in blue color.

Fig. S1

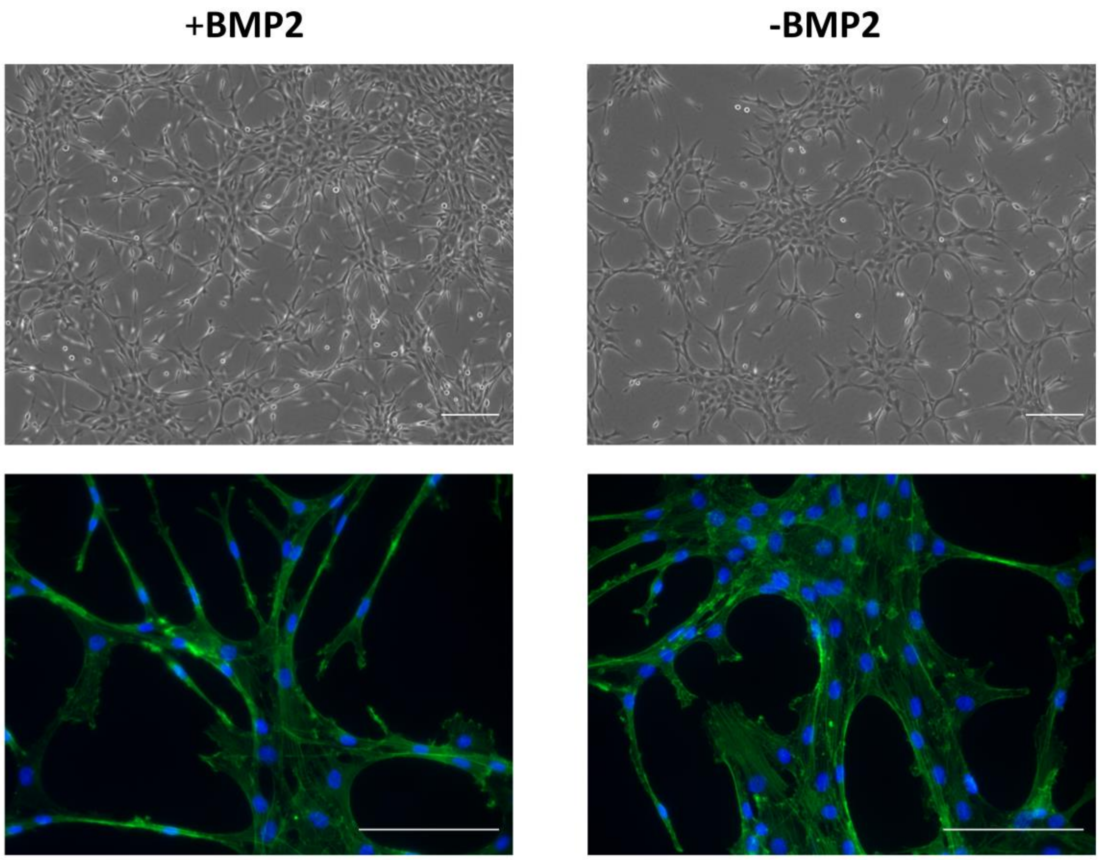

Figure S2 Transmission electron microscopy. Example shown from one donor (D8). The presented selection is a frontal sample from the middle of the cartilage disc (left). Cross section of the cartilage disc (center) showing sites of TEM images of the matrix (right, scale bar = 500 nm).

Fig. S2

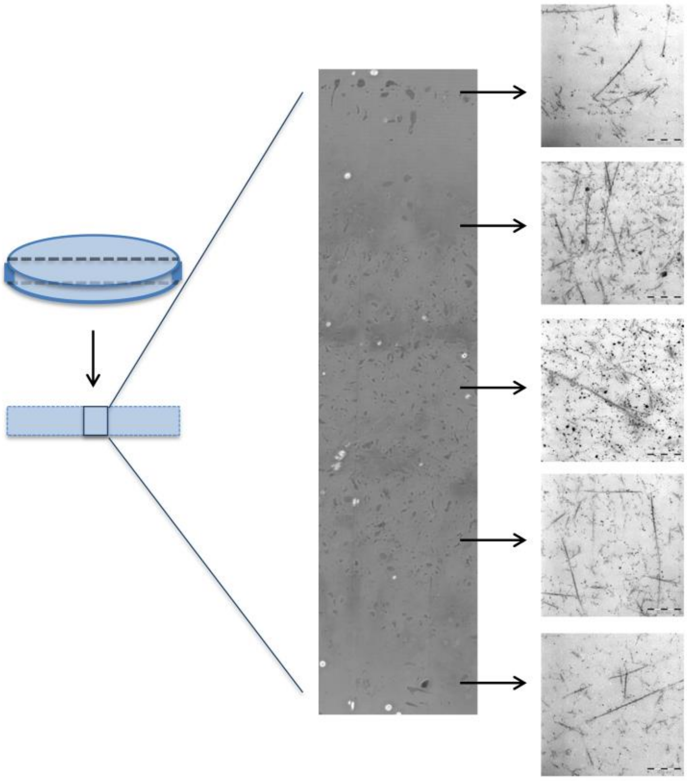

Figure S3 Second harmonic generation microscopy. Examples from cartilage discs based on chondrocytes from 2 donors (D5, D8). The section (left) is a frontal sample from the middle of the cartilage disc at low magnification (scale bar = 200 µm) showing sites of selected high-resolution images (right, scale bar = 50 µm). Fibrillar collagen presents in red, nuclear staining with DAPI presents in green.

Fig. S3

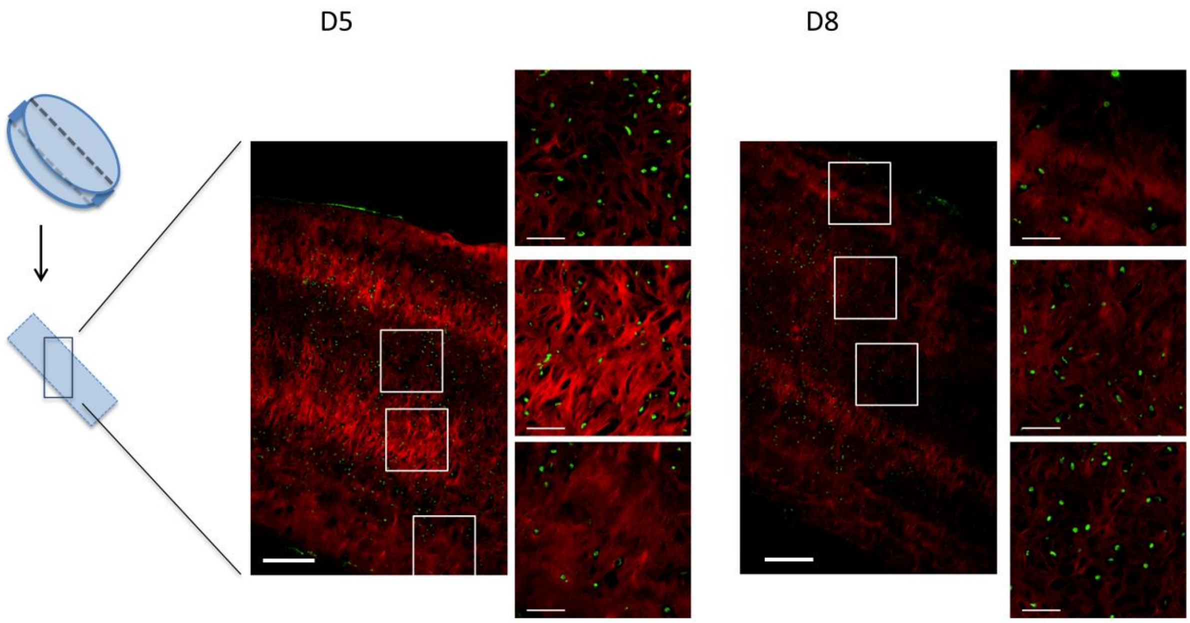

Supplement: sj-pdf-1-car-10.1177_19476035211007923 – Supplemental material for Scaffold-Free Engineering of Human Cartilage Implants [file sj-pdf-1-car-10.1177_19476035211007923.pdf]
